# A Model In Vitro Study Using Hypericin: Tumor-Versus Necrosis-Targeting Property and Possible Mechanisms

**DOI:** 10.3390/biology9010013

**Published:** 2020-01-07

**Authors:** Yue Li, Shuncong Wang, Yuanyu Zhao, Hexige Saiyin, Xiaoyan He, Juanzhi Zhao, Ling Li, Ali Talebi, Gang Huang, Yicheng Ni

**Affiliations:** 1 Shanghai Key Laboratory of Molecular Imaging, Shanghai University of Medicine and Health Sciences, Shanghai 201318, China; liy_16@sumhs.edu.cn (Y.L.); hexy@sumhs.edu.cn (X.H.); 2 KU Leuven, Campus Gasthuisberg, Faculty of Medicine, 3000 Leuven, Belgium; shuncong.wang@kuleuven.be (S.W.); ali.talebi@kuleuven.be (A.T.); 3 Technology Center of Chongqing Entry-Exit Inspection and Quarantine Bureau, Chongqing 401147, China; zhaoyuanyu414@163.com; 4 Laboratory of Translational Medicine, Jiangsu Province Academy of Traditional Chinese Medicine, Nanjing 210028, China; zhaojuanzhi1990@163.com (J.Z.); liling3208@163.com (L.L.); 5 State Key Laboratory of Genetic Engineering, School of Life Sciences, Fudan University, Shanghai 200433, China; saiyin@fudan.edu.cn

**Keywords:** hypericin, necrosis-avidity, liver cancer, fluorescence, confocal microscopy and mechanism

## Abstract

Hypericin (Hyp) had been explored as a tumor-seeking agent for years; however, more recent studies showed its necrosis-avidity rather than cancer-seeking property. To further look into this discrepancy, we conducted an in vitro study on Hyp retention in vital and dead cancerous HepG2 and normal LO2 cell lines by measuring the fluorescence intensity and concentration of Hyp in cells. To question the DNA binding theory for its necrosis-avidity, the subcellular distribution of Hyp was also investigated to explore the possible mechanisms of the necrosis avidity. The fluorescence intensity and concentration are significantly higher in dead cells than those in vital cells, and this difference did not differ between HepG2 and LO2 cell lines. Hyp was taken up in vital cells in the early phase and excreted within hours, whereas it was retained in dead cells for more than two days. Confocal microscopy showed that Hyp selectively accumulated in lysosomes rather than cell membrane or nuclei. Hyp showed a necrosis-avid property rather than cancer-targetability. The long-lasting retention of Hyp in dead cells may be associated with halted energy metabolism and/or binding with certain degraded cellular substrates. Necrosis-avidity of Hyp was confirmed, which may be associated with halted energy metabolism in dead LO2 or HepG2 cells.

## 1. Introduction

Hypericin (Hyp), a naturally derived small molecular fluorescent compound from St. John’s Wort, can be synthesized with anthraquinone derivative emodin. Previous studies showed that Hyp can be utilized as a marker for cancer [1,2]. Moreover, it can be used as a photosensitizer in photodynamic therapy for the treatment of superficial tumors due to this tumor-seeking property [3]. However, more recently, it was found that retention of Hyp in tumors could be attributed more to necrosis-avidity as its newly discovered property rather than to its assumed tumor targetability [4,5,6,7,8,9,10,11,12]. This finding complies with the fact that 30–80% of the solid tumors are accompanied by necrosis [13]. In addition, the necrosis avidity of Hyp was also confirmed by in vivo tumor models, with the higher retention in necrotic tumor than that in vital tumor [14,15]. However, the necrosis avidity demonstrated solely in animal levels is not persuasive enough due to the existence of sporadic necrosis inside tumor lesion, and therefore, an in vitro study is necessitated. So far, no study is available to answer this question. Therefore, we have elaborated this topic by in vitro experiments of Hyp accumulation in dead and vital neoplastic and nonneoplastic cells.

In addition, previous publications have demonstrated the applications of necrosis-avidity of Hyp in the visualization of acute myocardial infarction and development of a broad-spectrum anticancer strategy, namely, OncoCiDia [16,17]. The microSPECT can detect the infarcted heart tissue due to selective retention of Hyp labelled with radioactive iodine-123 in vivo [16]. This necrosis-avidity can be further explored for a novel dual-targeting anticancer approach where a vascular disrupting agent first shuts down cancer blood supply to induce (incomplete) tumor necrosis, which is followed by subsequent radioiodinated Hyp (131I-Hyp) to eliminate remaining cancer cells by emitted high-energy β-particles. Further improvement of these translational applications could be realized by structural optimization of the necrosis-avid agent based on the understanding of its mechanisms of action. However, to date, the mechanisms underlying the necrosis-avidity of Hyp continue to be debated. Although passive diffusion of the unbound chemical may also exist, carrier-mediated transcytosis was considered as a dominant form in Hyp transportation [18,19]. However, this hypothesis is contradictory with the characteristics of necrosis, i.e., stagnated energy-producing metabolism, which is crucial for carrier-mediated transcytosis. Previously, the necrosis-avidity of Hyp was found to be associated with integration of DNA or histone due to charge attraction, which is contradictory to our preliminary finding that the fluorescence emitted by Hyp mainly accumulate in cytoplasm [20,21]. Additionally, ^64^Cu-Bis-DOTA-conjugated Hyp showed high binding affinity to phosphatidylethanolamine (PE) and phosphatidylserine (PS), which mainly locate on the inner layer of plasma membrane in normal condition and will be transferred to the outer layer after cell death, suggesting that the Hyp merely binds with exposed PE and PS in dead cells rather than enters the vital cells [22,23].

**Figure 1:**
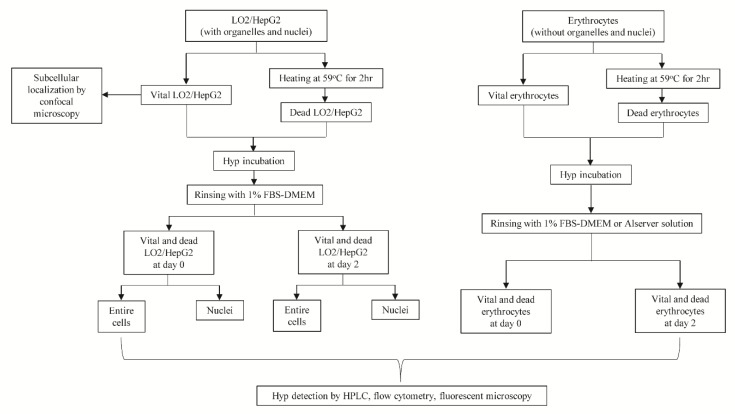
Flow diagram of protocols for the current in vitro study. Note: LO2 is a normal liver cell line, whereas HepG2 is a human-derived hepatocellular carcinoma cell line. FBS-DMEM is the Dulbecco’s Modified Eagle’s Medium mixed with fetal bovine serum.

Here, the current in vitro study (Figure 1) was conducted to address the following questions: (1) Is Hyp a cancer-specific or necrosis-specific agent? (2) Where is subcellular localization of Hyp? and (3) What is the mechanism of transmembrane transportation of Hyp? Specifically, we compared the accumulations of Hyp in LO2 (nonneoplastic hepatocytes) and HepG2 (hepatocellular carcinoma cells) cell lines in both vital and dead forms and preliminarily explored the mechanisms by detecting the subcellular localization of Hyp. Elucidation of these questions not only prompts the optimization of clinical translation of necrosis-avidity-based anticancer strategies but also deepens understandings of other diseases sharing necrotic features.

## 2. Materials and Methods

### 2.1. Chemicals

Hyp, Alsever solution (A3551), Thiazolyl Blue Tetrazolium Bromide (MTT, M2128), and other reagents, unless specified, were purchased from Sigma-Aldrich (St. Louis, MO, USA). Nuclear Extraction Kit (ab113474) was obtained from Abcam (Cambridge, UK). ER-Tracker™ Green dye (E34251), MitoTracker™ Green FM (M7514), and LysoTracker™ Green DND-26 (L7526) were purchased from Invitrogen (Carlsbad, CA, USA). Hyp was dissolved in dimethyl sulfoxide (DMSO) for a preparatory solution. The solution with a final concentration of 1 μM was added in the culture medium.

### 2.2. Cell Culture

Normal liver cell line (LO2) and hepatocellular carcinoma cell line (HepG2) were obtained from the cell bank of Chinese Academy of Sciences (Shanghai, China) and cultured in Dulbecco’s Modified Eagle’s Medium (DMEM), supplemented with 10% bovine fetal serum, L-glutamine and high glucose (4500 mg/mL), 1% penicillin/streptomycin, and 1% sodium pyruvate. Erythrocytes from a 41-year old healthy male were cultured in either the aforementioned culture medium or Alsever solution, which has been used to preserve erythrocytes. Dead cells were induced by heating at 59 °C for two hours and confirmed by trypan blue staining. Most cells were incubated at 37 °C in the dark with 5% CO_2_ and 95% humid atmosphere except for erythrocytes, which are cultured in Alsever solution at 4 °C to mimic clinical preservation conditions.

#### 2.2.1. Intracellular Accumulation for Hyp

Both LO2 and HepG2 cell lines were equally divided into two portions, with one portion being induced to death (Figure 1). In a pilot study by MTT cell viability assay, no effect on the number of the viable cells was observed when incubated at 37 °C with 1 μM Hyp for one hour or two days. Subsequently, both vital and dead cells were co-cultured with 1 μM Hyp in complete medium for one hour at 37 °C and harvested after rinsing. Similarly, groups of both vital and dead cells were co-cultured for two days and harvested, with rinsing by 1% FBS-DMEM six times per day. Erythrocytes can serve as a model for studying Hyp accumulation in cells without organelles and nuclei to test the hypothesis that Hyp integrates with DNA or organelles for necrosis avidity. Except for culture temperature and culture medium, the experimental conditions for erythrocytes are similar to those in LO2 cells or HepG2 cells. Nuclear extraction was performed with the extraction kit. The fluorescence of Hyp in cells and extracted nuclear were measured by fluorescent microscopy and quantitatively by flow cytometry. Since the intensity of fluorescence of Hyp may not correlate well with corresponding concentration, we performed reverse phase high-performance liquid chromatography (RP-HPLC) for accurate quantification [18,24]. Each type of samples was treated in triplicate.

#### 2.2.2. Fluorescent Microscopy

LO2, HepG2, their nuclear, and erythrocytes were observed in the same light intensity by fluorescence microscopy (Axio Vert.A1, Carl Zeiss Microscopy GmbH, Ostalbkreis, Germany) equipped with an excitation light source (X-Cite^®^ series 120Q, Lumen Dynamicse Group, Inc., Mississauga, ON, USA). Zeiss filter set 13 was used for visualization of the red fluorescence Hyp emitted after excitation, and the emission filters were set as 472/20 and 505–530 nm respectively. Images were captured with the same exposure time by a digital camera (AxioCam HRc, Carl Zeiss Meditec, AG, Jena, Germany) and processed with software (Zen 2011 Blue Edition, Carl Zeiss, Ostalbkreis, Germany).

#### 2.2.3. Flow Cytometry

The fluorescence intensity of Hyp in cell or nuclei was acquired using a FACSCanto^TM^ flow cytometer (BD Biosciences, San Jose, CA, USA). The 488-nm line of Argon laser was used for excitation. Hyp fluorescence emissions were measured in the PI/PE channel (channel B2 585/20 nm bandpass filter). Data were acquired using BD FACSDiva software (BD Bioscience, San Jose, CA, USA) and analyzed using WinMDI (version 2.9) to figure out mean fluorescence intensity (MFI).

#### 2.2.4. HPLC

The number of cells or nuclei per loaded sample was counted and unified by hemocytometry before measurement of the concentration of Hyp in either cells or nuclei by RP-HPLC. The preparation of samples includes extraction with acetone, drying by nitrogen, and dissolution by DMSO. The chromatographic analysis was performed using a Waters 2695 pump connected to a GRACE Alltima™ C18 analytical column (Millipore, Billerica, MA, USA) (250 mm × 4.6 mm, 5 μm), a Waters 2998 PDA detector (254 nm, Millipore, Billerica, MA, USA), and a Waters 2475 Muli λ fluorescence detector (Millipore, Billerica, MA, USA). The chromatographic conditions were as follows: flow rate of 1 mL/min; sample injection volume of 30 μL; column temperature of 30 °C; mobile phase A of methanol; and mobile phase B of 5 mM ammonium acetate. The gradient profile was optimized as the following: 0–5 min, 15% B; 6–12 min, 10% B; and 13–20 min, 15% B. For Hyp, the fluorescent detector was set with excitation and emission wavelengths of 315 and 590 nm, respectively. Data acquisition and processing were performed using Empower™ 3 Software (Shimadzu, Japan). The concentration of Hyp was calculated by the standard concentration-fluorescence intensity curve, and based on these calibration curves, cell count and negative counts can be calculated.

#### 2.2.5. Co-Localization of Hyp and Organelles

Confocal microscopy of both LO2 and HepG2 were performed. Cells were incubated with Hyp (1 μM) for 1 h at 37 °C, after which the cells were rinsed three times and incubated with dyes for different organelles (LysoTracker^®^ Green DND-26 at 50 nM, λabs = 504 nm, and λem = 511 nm; MitoTracker^®^ Green FM at 250 nM, λabs = 490 nm, and λem = 516 nm; or ER-Tracker™ Green dye at 1 μM, λabs = 504 nm, and λem = 511 nm) for 30 min at 37 °C and then 4′,6-diamidino-2-phenylindole (DAPI) (1 μg/mL, λabs = 340 nm, and λem = 454 nm) for 15 min. Confocal images were acquired with a Leica SP8 (Leica Microsystems, Wetzlar, Germany) confocal laser scanning microscope and processed with Fiji image J software (NIH, Bethesda, MD, USA).

#### 2.2.6. Cell Fixation and Incubation with Hyp

Cell fixation, which disables transmembrane receptor-mediated endocytosis, was performed to validate the possible involvement of passive diffusion. LO2 or HepG2 cells were first fixed in formalin for 10 min at room temperature and then incubated with Hyp (1 μM) for either one hour or 20 h. Afterwards, the images for Hyp location were taken by fluorescence microscopy after rinsing for three times.

#### 2.2.7. Statistical Analyses

All statistical analysis was performed with SPSS 24.0 (IBM Corp. Released 2016. IBM SPSS Statistics for Windows, Version 24.0. Armonk, NY, USA). Numerical data were expressed as mean ± standard error of the mean and were compared using one-way ANOVA analysis or Student’s *t* test. If significant, the differences between the means were assessed by the least-significant difference test. A *p*-value of less than 0.05 was considered as statistically significant.

## 3. Results

### 3.1. Accumulation of Hyp in Cells

**Figure 2:**
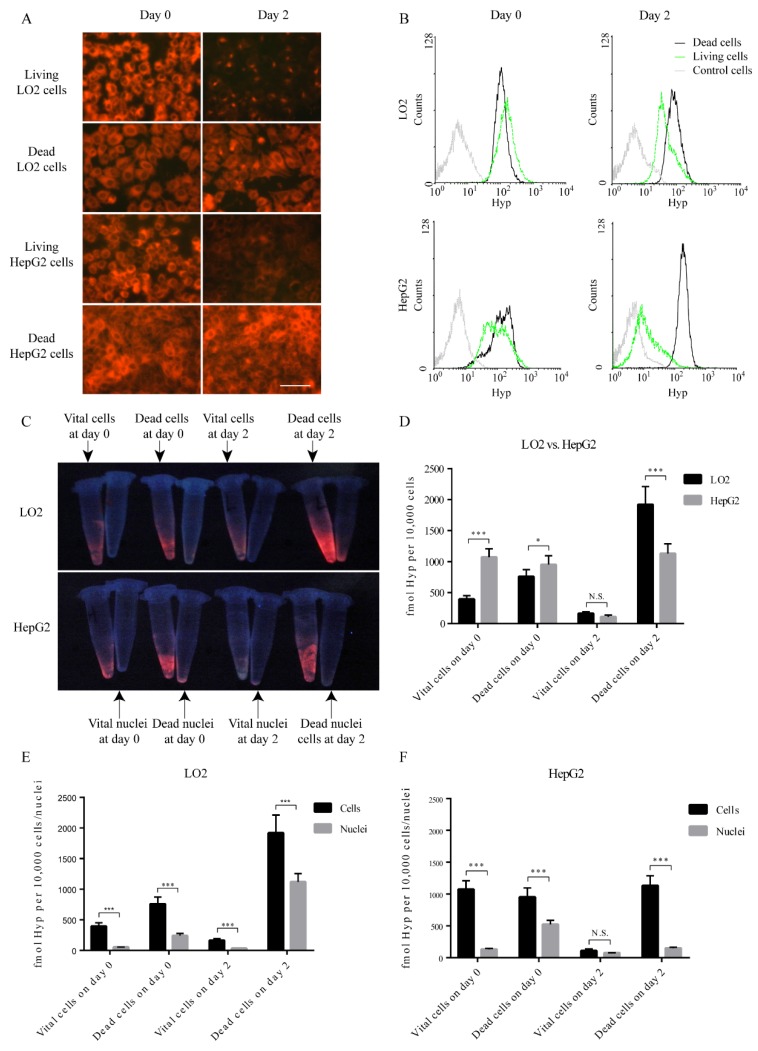
Hyp fluorescence in vital or dead neoplastic and nonneoplastic hepatocytes and paired nuclei. (**A**) The red fluorescence of Hyp observed by fluorescence microscopy in neoplastic and nonneoplastic cells at early and late stages. Scale bar = 50 µm. (**B**) Flow-cytometry histograms of Hyp fluorescence in control cells, vital cells, and dead cells. The control cells were not incubated with Hyp. (**C**) The fluorescence image of Hyp in cells and nuclei. (**D**) Comparison of Hyp concentration by RP-HPLC between LO2 and HepG2 cells (N = 3, Student’s *t* test). (**E**) Comparison of Hyp concentration measured by RP-HPLC on either cells or nuclei in LO2 cells (N = 3, Student’s *t* test). (**F**) Comparison of Hyp concentration measured by RP-HPLC on either cells or nuclei in HepG2 cells (N = 3, Student’s *t* test).

Before incubation of either normal cells or cancer cells with Hyp, we tested the safety profile of different Hyp concentrations by MTT tests and found that co-culturing cells with 1 μM Hyp did not alter the cell viability. Intracellular fluorescence was observed immediately after incubation (day zero) in both vital and dead LO2 and HepG2 cell lines, suggesting a paucity of selectivity in the retention of Hyp in the early phase (Figure 2A). After incubation with different concentrations of Hyp for one hour, LO2 cell line consistently shows stronger fluorescence than that of HepG2 cell line (Figure S1). However, the fluorescence dimmed after incubation for two days in vital cells (LO2 and HepG2 cells) but not in dead counterparts (*p* < 0.001). This trend was quantitatively validated by flow cytometry (Figure 2B). Specifically, the MFI ratios in dead cells over vital cells increased from 0.71 to 1.82 in LO2 cells and from 1.27 to 14.21 in HepG2 cells (Table 1). The MFI in vital HepG2 cells on both day zero and day two (93.30 and 12.84) are consistently lower than in LO2 cells (149.36 and 46.59).

### 3.2. Hyp Accumulation in Nuclei

**Figure 3:**
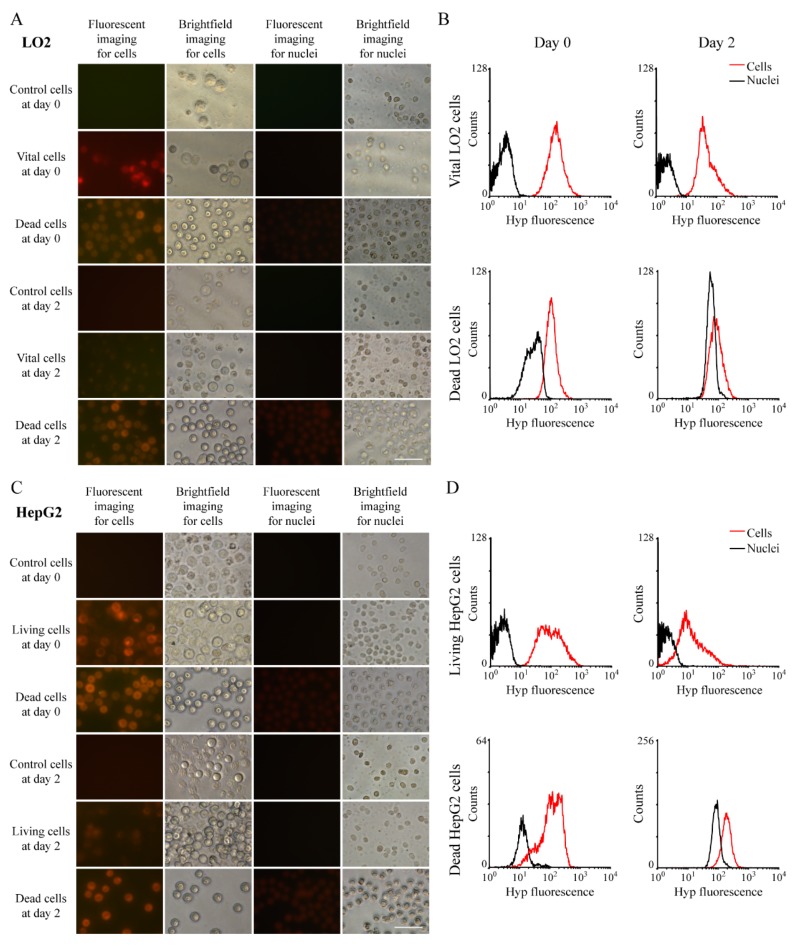
Hyp concertation in cells and paired nuclei: (**A**) Hyp fluorescence under ultraviolet light or white light of LO2 cells or nuclei extracted from paired cells. Scale bar = 50 µm. (**B**) Measurement of Hyp concentration in LO2 cells and their nuclei by flow cytometry. (**C**) Hyp fluorescence under ultraviolet light or white light of HepG2 cells or nuclei extracted from paired cells. Scale bar = 50 µm. (**D**) Measurement of Hyp concentration in HepG2 cells and their nuclei by flow cytometry.

To clarify the retention of Hyp in nuclei, we equally divided cells into two groups, with nuclei in one group being extracted. After the similar incubation protocol, we collected these cells and nuclei, observed them under fluorescence microscopy, and detected their concentration via RP-HPLC (Figure 2C–F). In both LO2 and HepG2 cell lines, Hyp concentration in whole cells is higher than that in nuclei in most settings except for viable HepG2 cells on day two (Figure 2E,F). On day zero and day two, concentration is generally lower in vital LO2 cells than that in vital HepG2 cells; however, this intercellular disparity in dead cells was reversed on day two (Figure 2D). In addition, we observed these samples under white light and under UV light for fluorescence in both cells and nuclei, which confirms our observations in RP-HPLC (Figure 3). In LO2 cell line, the fluorescence of nuclei from both vital and dead cells is significantly lower than those from corresponding entire cells in day zero and day two, and this comparison was quantified by flow cytometry (Figure 3A,B). A similar trend can also be seen in HepG2 cells line (Figure 3C,D).

### 3.3. Subcellular Hyp Fluorescence Distribution

**Figure 4:**
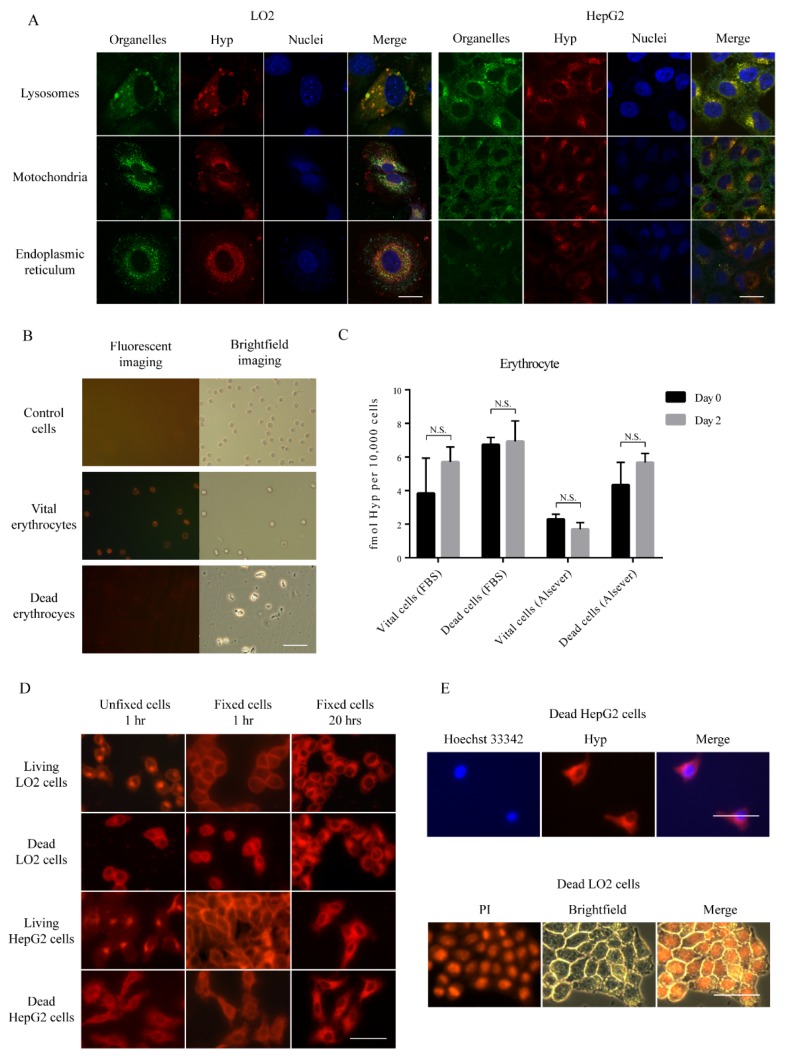
Subcellular localization of Hyp: (**A**) Fluorescence confocal microscopy of subcellular localization of Hyp in vital cells. Scale bar = 20 µm. (**B**) Visualization of erythrocytes under ultraviolet light or white light. Scale bar = 50 µm. (**C**) Comparison of concentration of Hyp in day zero and day two in erythrocytes. Data are expressed as the mean ± standard error (N = 3). (**D**) Subcellular localization of Hyp in fixed vital or dead cells. Scale bar = 50 µm. (**E**) Representative images of dead cells after staining for DNA by Hoechst 33342 and propidium Iodide (PI) or after incubating with Hyp.

To clarify the subcellular localization of Hyp, we performed confocal microscopy after staining for lysosomes, mitochondria, and endoplasmic reticulum in vital cells. Hyp co-located mostly with lysosomes rather than in mitochondria or endoplasmic reticulum in both vital LO2 and HepG2 cells (Figure 4A). In terms of erythrocytes, as the fluorescence was too week on day two, images for Hyp were taken only from the erythrocytes incubated in FBS-DMEM solution at day 0 and the signals were too low to be detected by the flow cytometer (Figure 4B). The concentration of Hyp in erythrocytes was extremely low, and no more difference was found in both time points (Figure 4C). Before fixation, Hyp fluorescence was mostly accumulated in the perinuclear region in vital cells, probably in lysosomes (Figure 4D). In contrast, a more diffuse pattern of Hyp accumulation was observed in unfixed dead cells. In fixed cells, after incubation of either one hour or 20 h, a similarly diffuse accumulation of Hyp was observed. Hyp marginally retain in DNA-rich region nuclei, after staining by either Hoechst 33342 or propidium Iodide (PI) (Figure 4E).

## 4. Discussion

The current study, for the first time, provides in vitro evidence supporting the necrosis-avidity of Hyp and excludes its tumor-seeking characteristics. In addition, transmembrane transportation of Hyp was mediated by passive diffusion, which helps explain the similar fluorescence intensity of Hyp in both vital and dead cells in early phase. Later on, Hyp within vital cells was taken up by lysosomes, where it is likely degraded and/or excreted, whereas the Hyp in dead cells retained intracellularly.

Our results showed that Hyp accumulates in both vital LO2 and HepG2 cells in early phase, as demonstrated by both fluorescence microscopy and quantitatively by HPLC. However, on day two, fluorescence was only retained diffusely in necrotic LO2 and HepG2 cells rather than in vital cells, and therefore, these results confirmed its necrosis-seeking ability rather than cancer-seeking ability. The diminished fluorescence in vital cells is mainly caused by the reduced intracellular quantity of Hyp rather than by being converted into non-fluorescent metabolite, as demonstrated by a previous study where intravenously injected Hyp can emit fluorescence even after being metabolized by liver [25]. Cell death, conventionally classified into programmed death and necrosis, is a common and essential part of the natural history of most cells, and it may be involved in the pathogenic process. Disruption of apoptosis, a major type of programmed death upon environmental signal or stress, may give rise to diseases. For instance, excessive neuron apoptosis may result in Alzheimer’s disease and a lack of apoptosis can be found in pro-cancerous cells leading to onset of cancer [26,27]. Necrosis is a passive form of cell death, which can be induced by ischemia, extreme physical and chemical conditions including high temperature, excessive toxins, absolute ethanol, and so forth. Necrosis-related diseases, mainly cardiovascular diseases with 17.9 million death each year, constitute a great number of patients’ death [28]. Studying cell death may help understand pathogenesis of cancer and Alzheimer’s disease and may develop theragnostic methods for diseases sharing necrotic features. Visualization of cell death and identification of dead cells are essential in bench studies. Currently available methods for visualization under intensive research include Annexin V-124, PGN650, ML-10, and so on [29]. Hyp, a natural derivative harbouring necrosis-avidity, has been explored extensively for its application in visualization of necrosis or necrosis-based anticancer strategy OncoCiDia [17]. Elucidation of the mechanism of necrosis-avidity at cellular level is a prerequisite for optimization of its application as an anticancer strategy.

Hyp, as a hydrophobic compound, can be transported into cells by passive diffusion in dead cells or living cells [30]. In the living cells, where ATP-dependent transportation is active, receptor-mediated transportation is another way for transmembrane transportation, which is more efficient than passive diffusion, as revealed by the slow cellular influx in the fixed cell in which receptor-mediated transportation is inhibited [19]. Afterwards, rapid decrease of Hyp in vital cells might be caused by degradation and/or excretion by lysosomes, which is also an ATP-dependent process and largely disabled in dead cells. These help explain that the fluorescence is highly concentrated in a focal point in unfixed cells whereas the fluorescence is diffuse and retained in fixed cell where energy metabolism is halted. However, another in vitro study shows that photodynamic therapy by Hyp exerts its therapeutic effect on glioma cells by the production of reactive oxygen species in Golgi bodies [31]. Concerning the role of low density lipoprotein (LDL) in the transmembrane transportation, some scholars claimed that LDL facilitates the subcellular localization of Hyp in lysosomes, whereas, other scholars hold the view that LDL may not participate in this process due to the fact that no reduced Hyp cellular accumulation was observed when LDL or acetylated-LDL (AcLDL) was added in cell culture medium [32,33,34]. In addition, it is unreported regarding the exact carriers that facilitate intracellular transportation of Hyp to the lysosome, and lipoprotein is a possible candidate because Hyp shows high affinity to both LDL and high-density lipoprotein (HDL) [25,35,36,37,38]. Albumin is also a possible transporter for Hyp [32]. We observed a higher proportion of albumin in necrotic tissue than in normal section of liver tissues by electrophoresis (data not shown). As albumin also tends to reside lysosome in vital cells, Hyp-albumin complex may consistently explain the diffuse distribution in dead cells and selective accumulation in lysosomes [35].

The intracellular binding target of Hyp remains largely unknown. PS is reported to locate primarily on the inner leaflet of the membrane in normal conditions with phosphatidylcholine (PC) on the outer leaflet [39]. The PS externalization occurred after cell death, and it is hypothesized that externalized PS is accountable for the high retention of Hyp in necrotic tissue due to the higher affinity of Hyp towards PS than PC [23]. However, this hypothesis is contradictory to our findings that Hyp content was even more in vital LO2 cells than that in dead cells on day zero. Furthermore, the Hyp selectively accumulated in the cytoplasm in dead cells and lysosomes in vital cells, where few PS are supposed to present. Additionally, the fluorescence in erythrocytes confirmed that neither PS nor PE on damaged membranes is likely bound by Hyp because only a marginal fluorescence can be observed in both vital and dead erythrocytes. Besides, unlike other visualizing agents by retention in nuclei due to the disability of efflux by P-glycoprotein, Hyp functions in a nuclei-independent manner [19]. Here, we observed marginal retention of fluorescence in nuclei in vital and dead cells and naked nuclei, a DNA-rich compartment. In addition, staining with PI shows that, after cell death, there is little DNA in the cytoplasm, where hyp mainly accumulate, and that these results together failed to support the hypotheses that Hyp intercalate with either DNA or histones by charge attraction [20]. 

In conclusion, the red fluorescent Hyp is characterized by stable and strong necrosis-avidity instead of tumor-seeking targetability. In vital LO2 or HepG2 cells, Hyp is likely to be transported into lysosomes by the carrier and discharged after degradation. However, Hyp retains in dead LO2 or HepG2 cells due to disenabled active excretion, leading to the observed diffuse distribution of the Hyp-carrier complex in the cytoplasm. Further researches are needed to clarify what molecules are involved in endocytic incorporation and transportation and what physicochemical interactions exist between Hyp and cellular substrates for the observed necrosis-avidity of Hyp.

## Figures and Tables

**Table 1:** The mean fluorescence intensity (MFI) of Hyp by flow-cytometric analysis in LO2 or HepG2 cells at early and late stages: The results are expressed as the mean ± standard error (N = 3, Student’s *t* test). Ratio: the ratio of MFI in dead cells to that in vital cells.

			LO2			HepG2	
		MFI	*p* Value	Ratio	MFI	*p* Value	Ratio
Day 0	Control cells	4.63 ± 0.2	<0.001	0.71	5.1 ± 0.07	<0.001	1.27
	Vital cells	149.36 ± 5.59			93.3 ± 0.27		
	Dead cells	106.78 ± 4.26			118.05 ± 0.5		
Day 2	Control cells	4.72 ± 0.05	<0.001	1.82	3.99 ± 0.03	<0.001	14.21
	Vital cells	46.59 ± 0.72			12.84 ± 0.16		
	Dead cells	84.9 ± 0.48			182.51 ± 0.27		

## Supplementary Materials

The following are available online at https://www.mdpi.com/2079-7737/9/1/13/s1, Figure S1: The fluorescence image of Hyp in either LO2 or HepG2 cell line after incubation with different concentrations of Hyp for one hour.
