# Supplementary material for: A Model In Vitro Study Using Hypericin: Tumor-Versus Necrosis-Targeting Property and Possible Mechanisms
Source: Biology (Basel). 2020 Jan 7;9(1):13. doi: 10.3390/biology9010013 (PMC7168897; doi:10.3390/biology9010013)
Supplement: Supplementary file 1 [file biology-09-00013-s001.pdf]

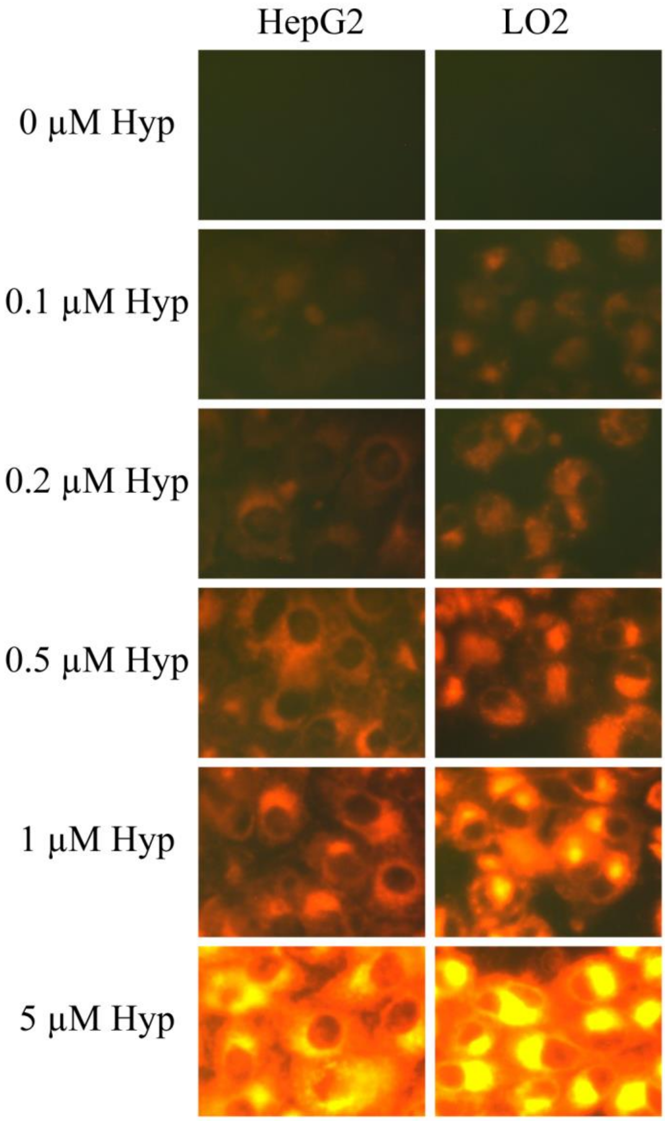

Figure S1. The fluorescence image of Hyp in either LO2 or HepG2 cell line after incubation with different concentrations of Hyp for one hour.
